# Despite an improved aerobic endurance, still high attrition rates in initially low-fit recruits—results of a randomised controlled trial

**DOI:** 10.1016/j.conctc.2020.100679

**Published:** 2020-11-28

**Authors:** I. Dijksma, W.O. Zimmermann, D. Bovens, C. Lucas, M.M. Stuiver

**Affiliations:** a Epidemiology and Data Science, Master Evidence Based Practice in Health Care, University of Amsterdam, Amsterdam, the Netherlands; b Physical Therapy Department, Medical Centre Airmobile Brigade, Royal Netherlands Army, Schaarsbergen, the Netherlands; c Department of Sports Medicine, Royal Netherlands Army, Utrecht, the Netherlands; d Uniformed Services University of the Health Sciences, Bethesda, MD, USA; e Department of Healthcare, Headquarters Royal Netherlands Army, Utrecht, the Netherlands

**Keywords:** Military trainees, Pre-training conditioning program, Cardiovascular endurance, Injury prevention, Complex system approach

## Abstract

**Background:**

Low baseline fitness of recruits entering basic military training (BMT) is associated with an increased risk of musculoskeletal injuries (MSIs) and attrition from training.

**Objective:**

To determine the effects of a pre-training conditioning program (PCP) on aerobic endurance, incidence of musculoskeletal injuries (MSIs), and attrition rates in BMT of a special infantry unit of the Netherlands Armed Forces.

**Participants:**

Recruits were considered eligible for this study when they were ‘low-fit’ at the start of BMT (time to complete a 2.7-km run ≥ 12′23″).

**Interventions:**

‘Low-fit’ recruits were deferred to a seven to twelve week—depending on the time between two consecutive training platoons—PCP consisting of functional training to improve several fitness domains. The control (CON) group started regular BMT without delay.

**Results:**

Forty-nine recruits were included in this study; 26 in the PCP-group and 23 in the CON-group. Recruits who followed the PCP started BMT with better aerobic endurance than the CON-group who started BMT immediately (2.7 km run timings: PCP 11′32″, CON 13′16″). The risk of dropout was lower in the PCP-group (incidence density ratio (IDR) 0.63, 95%CI 0.32; 1.26), but dropout due to training-related MSIs was more frequent (IDR 1.23, 95%CI 0.32; 4.76 (per-protocol 0.94, 95%CI 0.24; 3.63), without statistically significant differences between the groups.

**Conclusion:**

Although a PCP was effective to improve aerobic endurance in low-fit recruits to return to meet pre-enlistment fitness criteria, we could not demonstrate an effect on dropout from military training.

**Trial registration:**

Dutch trial register Trial NL6791 (NTR6977) https://www.trialregister.nl/trial/6791.

## Highlights

• Low baseline fitness of recruits is associated with musculoskeletal injuries and dropout from military training.
• A pre-training conditioning program was capable of improving physical fitness in low-fit recruits.
• However, dropout from training remained high, perhaps due to common other causes.
• Future research should focus on identifying these physical, social and mental factors, and their interactions.

## 1 Introduction

Poor physical fitness has shown to be strongly associated with an increased risk of training-related musculoskeletal injuries (MSIs) in military trainees [[1], [2], [3], [4], [5]]. In particular, there is strong evidence that poor performance on a timed run test with a fixed distance is a predictor for such injuries [6]. MSIs are among the main causes for dropout from basic military training (BMT) [7,8].

In the Royal Netherlands Army (RNLA), pre-enlistment fitness tests are used to select eligible recruits for BMT. This training course lasts 24 weeks, including basic training and advanced airmobile infantry training. Several months may pass between testing and the first day of BMT, and recruits who initially pass the fitness tests regularly decline in fitness during these months. Unpublished data from 2015 to 2017 showed that of a total of 734 recruits of BMT of the Dutch Airmobile Brigade, 18% no longer met physical fitness criteria in week one of BMT. In these recruits, the risk of attrition due to MSIs was 4.3 times higher in recruits with a time at or above 12 min on a set distance run (2.7 km) versus those who finished the run below 12 min (26% vs 6% dropout).

In an effort to reduce MSI's and drop-out due to low starting fitness, we developed a pre-training conditioning program (PCP) for low-fit recruits in the Airmobile BMT. To our knowledge, few studies have been performed to estimate the effects of such pre-training conditioning programs for low-fit recruits [[9], [10], [11]]. Two of these studies had a large sample size and suggested positive effects on both physical fitness as well as the risk of dropout from training [9,10]. However, those studies were observational by design, and therefore at risk for several sources of bias [12]. Therefore, we introduced the PCP program using a randomised controlled design. The following study questions were addressed: 1) Do low-fit recruits, who followed the PCP, show better cardiovascular endurance post-intervention and at mid-term BMT than recruits following the regular procedure? 2) Is the risk of attrition due to overuse injuries in low-fit recruits who followed the PCP lower than in those who followed the regular procedure? 3) What barriers and facilitators are identified for structural implementation of a PCP for low-fit recruits at the start of the BMT?

## 2 Methods

### 2.1 Setting and participants

The source population for the study was formed by Airmobile recruits of the Airmobile Brigade of the RNLA. The trial was conducted at the Airmobile Training Centre, Schaarsbergen, The Netherlands. Participants were enrolled at the start of their initial military training and monitored until completion of the BMT, or until dropout from training. Prior to participating in initial military training, all applicants must pass a three-day functional physical and mental test and a centralized medical screening. Recruits were considered eligible for this study when they were ‘low-fit’ at the entry of BMT (week one BMT). Recruits were considered low-fit when they completed the 2.7-km run test in ≥12′23”. Recruits who completed the 2700 m run test <12′23″ were admitted to the regular training course and were not included in this study; when aerobic endurance at entry BMT is sufficient, the goal is to maintain that level of fitness during the course rather than to improve further in that domain.

### 2.2 Design

The Medical Research Ethical Committee of the University Medical Centre Utrecht, The Netherlands confirmed that the national Medical Research Involving Human Subjects Act did not apply to this study (protocol number: 17–631/C) and waived the study from formal approval. The study was conducted in accordance with the Declaration of Helsinki. The trial was registered a priori in The Netherlands National Trial Register (https://www.trialregister.nl/trial/6791).

A detailed description of the study design has been published previously [13]. Briefly, this study employed a Zelen's design using a double consent procedure [14]. After a physical fitness test in week 1 of the BMT, but prior to being informed about the study and signing informed consent, eligible recruits were randomised to one of two groups; the intervention group (PCP), or the control group (CON: regular procedure Airmobile BMT).

### 2.3 Interventions

The PCP was conducted by military staff and instructors of the Airmobile Training Centre, sports instructors and other military experts (i.e. embedded monitors, medical staff). A complex system approach was applied to cover several domains of MSI prevention other than physical fitness (i.e. mindset, health accountability) and to optimize intervention circumstances (i.e. training staff support) [[15], [16], [17]]. The PCP filled the gap between two successive cohorts, at which the period varied from seven to twelve weeks. The physical fitness training program focussed on functional training to improve mobility, power, agility, strength, and cardiovascular endurance. Cardiovascular endurance was targeted both by endurance training and high-intensity interval training. Training intensity was individually adapted for heart rate (HR) zones. HR was monitored by a Polar H10 (Polar, Kempele, Finland) heart rate monitor.

The CON-group received the standard physical training program, supervised by designated sports instructors, during the whole BMT. This program included running, callisthenics, obstacle course, strength circuits, military self-defence, wall climbing, and rope climbing. Recruits in the CON-group were briefly informed regarding nutrition and recovery, conform usual practice.

### 2.4 Outcome measures

The primary outcome measure was time to complete a 2.7-km run mid-term BMT. There were three military physical fitness test sessions: baseline measurement (B0), post-intervention measurement and week one BMT for PCP group (W0), control group measurement at time point in weeks since start intervention (W9, note that the timing of this measurement varied according to the duration of the PCP (7, 9, 12, 10 weeks), measurement at mid-term BMT (W12, week 12 for both groups). Secondary outcomes included MSI incidence per body region, self-reported (free from-)injury states (5-point Likert scale), lost training days, attrition rates, and intervention evaluation. The intervention was evaluated by an anonymised and confidential survey administered to recruits and training staff.

### 2.5 Sample size calculation

A priori sample size calculations indicated that 37 participants (i.e. approximately 19 per group) were required to provide 80% power to detect a clinically meaningful difference of 35 s (standard deviation 30 s) on the 2.7-km run test at mid-term BMT, with alpha set at 5% and taking a 40% dropout rate into account [2,6].

### 2.6 Statistical analysis

Statistical analysis was performed using R statistical software (version 3.6.1) in RStudio. We used the intention-to-treat principle, where all participants were included in the final analysis according to their randomised allocation. Additionally, we performed a per-protocol analysis based on received intervention.

Baseline characteristics were summarised using descriptive statistics. Baseline comparability of clinical and sociodemographic characteristics was checked. Within subjects changes over time were tested using non-parametrical Wilcoxon's signed rank tests. A linear mixed-effects model with a random intercept and a group-by-time interaction term was used to test the primary outcome. Mean difference in change of time to complete the 2.7-km run, from selection to mid-term, was expressed using a standardised effect size, Cohen's *d* (0.2 small effect, 0.5 medium effect, 0.8 large effect) [18]. Clinical reported MSI incidence was described as incidence rates per person-year (py) and incidence density rates (IDR) between the groups. Attrition rates and lost training days were also expressed as IDR with a 95% confidence interval (95% CI). We preferred to perform a complete case analysis and refrain from data imputation because of the high dropout rates (see results). Statistical significance was set at *p* < 0.05.

Self-reported (free from-)injury states were descriptively reported throughout the intervention period. Noted barriers and solutions to those barriers (including five statements to be scored on a 5-point Likert scale and one open question in which participants were asked to provide a brief reflection on the program as a whole), were summarised descriptively.

## 3 Results

**Fig. 1:**
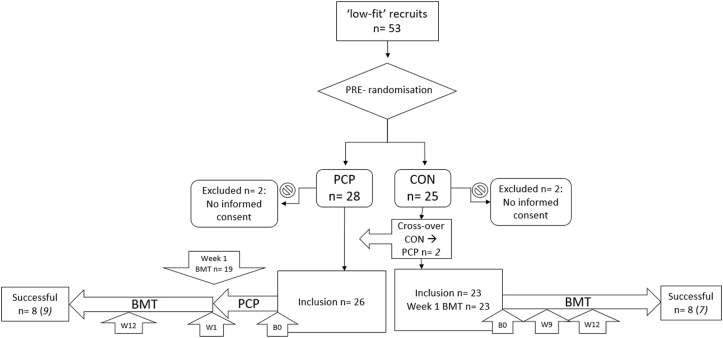
Flow chart of participants Legend: PCP = pre-training conditioning program group, CON = control group, BMT= Basic military training, B0 = baseline measurement, W1 = post-intervention measurement and week one BMT for PCP group, W9 = control group measurement at time point in weeks since start intervention (note that the timing of this measurement varied according to the duration of the PCP (7, 9, 12, 10 weeks), W12 = measurement at mid-term BMT (week 12 for both groups), corrected for crossover 9 were successful in the PCP-group, compared to 7 in the CON-group.

Fifty-three recruits originating from four consecutive training cohorts completed the 2.7-km run in ≥12′23″ and were consequently considered low-fit at the start of BMT and pre-randomised. All recruits included in this study were male. Two were not able to complete the run test at all at baseline, and 14 completed the run in >13′47”. Three decided to leave BMT before being informed about the study (CON:1, PCP:2), and one did not provide informed consent (CON:1). Two requested crossover from CON to PCP, of which one completed BMT. See Fig. 1 for flow chart of participants and Table 1 for participant demographics. The duration of the four consecutive intervention periods (PCP) was seven, nine, twelve and ten weeks, respectively.

**Table 1:** Participant demographics.

	Pre-training conditioning group	Control group
Baseline (W0)	n = 26	n = 23
**Age (med, IQR)**	20 (19–21)	20 (19–21)
**Height, cm (mean** ± **SD)**	180.5 ± 7.16	181.5 ± 5.63
**Weight, kg (med, IQR)**	79.5 (68.8–90.6)	82.9 (74.6–88.7)
**Bodyfat % (med, IQR)**	16.6 (9.6–20.1)	17.7 (13.8–19.9)
**2.7-km run time (mean** ± **SD)**	13′26” ± 54.3” ‡	13′16” ± 58.5″

Legend: BMT= Basic military training, med = median, IQR = interquartile range, SD = standard deviation, km = kilometre, *pro-agility test, ^60-m sprint test, † average of two measurements, ‡n = 2 did not finish 2.7-km run.

### 3.1 Physical fitness

Due to logistic reasons in BMT, and beyond our control, control group measurements of physical fitness at time point in weeks since start intervention (W9) were cancelled, and fitness measurements at mid-term BMT (W12) were cancelled for the last cohort of the PCP-group. Therefore, there were 36 missing observations (73%) at W12 of which 32 (65%) from recruits who dropped out from training before the mid-term test took place. Thus, only 5 PCP and 8 CON participants could be included in the analysis of the primary outcome. After completing the PCP, (W1 post-intervention) two recruits were still not able to achieve fitness criteria (running 12′24″ and 12′31″, respectively). One was discharged as per personal request, one was retained by recommendation of the instructors and completed BMT.

**Fig. 2:**
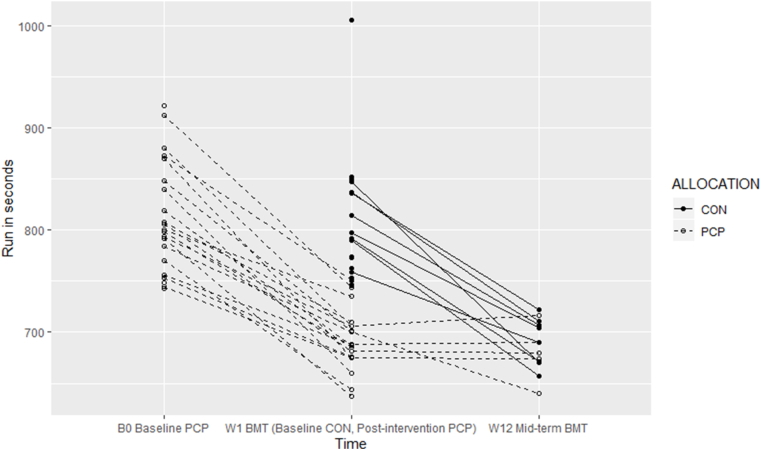
Cardiovascular endurance Legend: BMT = basic military training, CON = Control group, PCP = Pre-training conditioning group. The time between baseline (B0) and W1 reflects the pre-training conditioning period for the intervention group, the time between W1 and W12 Mid-term BMT reflects basic military training for both the control group (who started BMT without delay) and the intervention group.

In the PCP-group, the 2.7-km timed run from baseline (W0) to post-intervention (W1) in recruits with complete follow up-data improved on average from 13′34″ to 11′32” (−121 s). The recruits for whom further follow-up data was available (n = 5) stayed relatively stable thereafter until mid-term BMT (on average 11′20″ at W12) (Fig. 2). In the CON-group, recruits with complete follow-up data improved on average from baseline (W0) 13′39″ to mid-term BMT (W12) 11′32” (−117.5 s). Thus, recruits in the PCP-group showed slightly more improvement on cardiovascular endurance, than recruits in the CON-group. All had a performance above 12 min mid-term BMT (W12). Linear mixed-effects analysis of the effect of the intervention on change in run timings from baseline to mid-term BTM showed a regression coefficient (β) of −24.64″ with a 95% CI of −54.45; 5.17 (per-protocol analysis β −24.42” (95% CI -54.52; 5.68). Cohens' *d* effect size of that change was 0.68 (95% CI -0.52; 1.82), indicating a medium, but not statistically significant effect, in favour of the PCP group.

### 3.2 Attrition rates

The total attrition rate was 67.3% with no significant difference between the groups (PCP 69%; CON 65%). In both the PCP-group as well as the CON-group, eight recruits completed the BMT. IDR for total dropout was 0.63, 95% CI 0.32; 1.26 (per-protocol 0.55, 95% CI 0.28; 1.09), in favour of the PCP-group. In the intention to treat analysis, dropout due to training-related MSIs was higher in the PCP group (IDR 1.23, 95% CI 0.32; 4.76) but it was lower in the per-protocol analysis (IDR 0.94, 95% CI 0.24; 3.63). In both groups, eight recruits withdrew voluntarily.

### 3.3 Musculoskeletal injuries

Nineteen recruits in the PCP-group and 12 recruits in the CON-group had a clinical reported MSI within the total training time (IDR 0.84, 95% CI 0.40; 1.72). In total, 63.3% of recruits had one MSI, 14.3% had two MSIs, and 2.04% had three MSIs. Most injuries were training-related (51.3%) and 25.6% occurred acutely. Leg and knee were the most common reported injury locations, followed by back and ankle. Fifty percent of acute injuries were ankle distortions. Table 2 presents the MSI incidence rates per person-year. We included all injuries in this analysis (acute and overuse injuries and first, second or third MSIs).

**Table 2:** Musculoskeletal injury incidence rate per person-year.

Incidence rate per person-year (95% Confidence Interval)			
Body region	Total sample	*PCP*	*CON*
**Leg/thigh**	0.52 (0.27–1.00)	0.44 (0.18–1.06)	0.67 (0.25–1.78)
**Knee**	0.46 (0.23–0.92)	0.44 (0.18–1.06)	0.50 (0.16–1.55)
**Ankle**	0.35 (0.11–1.07)	0.18 (0.04–0.71)	0.17 (0.02–1.19)
**Back**	0.35 (0.11–1.07)	0.09 (0.01–0.63)	0.33 (0.08–1.34)
**Foot**	0.29 (0.11–0.77)	0.26 (0.09–0.82)	0.17 (0.02–1.19)
**Hand**	0.12 (0.02–0.82)	0	0.17 (0.02–1.19)
**Hip**	0.12 (0.03–0.46)	0.18 (0.04–0.71)	0
**Neck**	0.06 (0.01–0.41)	0	0

Legend: CON = Control group, PCP = Pre-training conditioning group.

Self-reported (free from-)injury states were comparable over the weeks and between the groups within a range of 60%–79% of recruits responding to be free from MSIs.

### 3.4 Lost training days

From 4140 training days in the PCP-group, 117 were lost. In the CON-group there were 78 lost training days out of 2186. This resulted in an IDR of 0.79, 95% CI 0.60; 1.05 (Fisher's exact p = 0.13). Per-protocol analysis showed significantly less lost training days in the PCP, IDR 0.74 95% CI 0.56; 0.99 (Fisher's exact p = 0.045).

### 3.5 Intervention evaluation

Twenty-six recruits filled in the survey. The survey revealed that 89% were grateful to get the chance to improve their fitness by the PCP before entering BMT, whereas 4% disagreed to this statement. According to the respondents, the program contained sufficient recovery time in between sports sessions (100%). However, 19% noted that the program did not offer sufficient military lessons besides sports sessions to keep them engaged. Recruits used the following terms most frequently to describe the PCP: innovative, instructional, active, unique chance, well-developed program, motivating, and preparative.

The majority of the training staff (89%) noted that they believed that the recruits in the PCP-group were able to work more consciously on their fitness compared to recruits in regular BMT. Fifty-six percent of the instructors believed that the PCP potentially contributed to improved success rates of the BMT. During the evaluation, the training staff also noted that in general, the consequences of not meeting fitness criteria at start of BMT should be emphasized stronger for new recruits, to improve mindset prior to BMT. No specific points of improvement for the PCP were suggested, but the experience with the PCP did prompt several suggestions to improve the BMT, in order to reduce MSIs and dropout from training: increase time in training (i.e. BMT from 24 to 28 weeks); shorten the time between pre-enlistment fitness test and the first day of BMT; a structured conditioning program should form a fundamental part (i.e. in the first two months) of BMT; the sports programs could be further individualised (as much as logistically and practically possible) for every recruit to work on his or her specific weaknesses during BMT.

## 4 Discussion

This study suggests that a pre-training conditioning program was capable of improving cardiovascular endurance in low-fit recruits, to again meet pre-enlistment fitness criteria for BMT. However, dropout from training remained high. The high dropout rates and cancelled measurements resulted in inconclusive evidence regarding both the primary and secondary outcomes.

Noteworthy, the PCP was able to improve aerobic endurance to again meet the minimum required level of fitness in week one—in those recruits who failed to maintain their fitness themselves—however, this did not result in a reduction of dropout from training in these ‘low-fit recruits’. Variables that influence the risk of dropout can be in several domains; social (e.g. supportive parents, group culture, military instructor), mental (e.g. self-confidence, grit, beliefs), and physiological (e.g. basic motor skills, (an-)aerobic endurance, strength, recovery) [17,19,20]. Although we used a complex system approach intended to optimize contextual factors (training staff support, mindset, health accountability), the main point of focus of the PCP was aerobic endurance (i.e. the physiological domain). Therefore, we did not extensively measure the effect of our approach on these contextual factors and it remains unclear how successful the PCP was in this regard.

Typically, during BMT, the job resources are low (e.g. recruits have low autonomy, and confidence and self-esteem are blunted by training staff), with very high job demands (e.g. vast progression in both military skills as well as tactical fitness), resulting in an imbalance between resources and demands and therefore high amounts of stress in recruits [21]. Ideally, through gradually upgrading physical fitness, without large increases in training load, one of the factors in the job demands can be reduced, theoretically resulting in better proportional overload and less physiological stress.

As expected by *adaptation* as a training principle, improvements in aerobic endurance manifested for the majority of the PCP participants in the pre-training phase and remained fairly stable thereafter, in the first half of military training—which is the intended outcome for recruits whose baseline aerobic endurance is sufficient [22]. Recruits in the CON-group showed similar improvements in aerobic endurance from week one until mid-term BMT, through the intensive and functional military training. This implies that mere improvements in physical fitness do not warrant implementation of a PCP, if there is not also a salutary effect of the PCP on dropout rates. On the other hand, if military sports instructors would take advantage of the improved fitness levels of post-PCP recruits, by individualizing training to target individual's specific strenghts and weaknesses rather than focussing on maintaining current fitness levels, improving physical fitness through PCP might still be of value [23]. This approach, which might lead to larger impact on MSI-related dropout, could be investigated in future studies.

Our study was not able to confirm neither to reject the findings of international colleagues. An observational study performed in the Singapore Armed Forces (n = 9109) suggested that a four to six-weeks conditioning program for unfit recruits before a twelve-week BMT resulted in a decreased risk of dropout (0.45, 95%CI 0.38; 0.62) [9]. Although the point estimate in our study pointed in the same direction of effect (i.e. a decreased risk after PCP), the effect was smaller and not statistically significant in the intention to treat analysis. A smaller, but randomised controlled study in 36 recruits in the Singapore Armed Forces studied the effect of a six-week conditioning program before a ten-week BMT in low-fit recruits on VO2max and 2.4-km run timings. The intervention group showed greater improvement on the 2.4-km run, however, this improvement was not statistically significant (p = 0.61) [10]. An observational study among 2072 American recruits showed that attrition rates and injury risk remained elevated in the preconditioning group, compared to the recruits who were initially fit and had no need for preconditioning [11]. This is what we also found in our study: low-fit recruits remained at increased risk of dropout and MSIs compared to their fitter counterparts, regardless of their improved physical fitness through the PCP. This suggests that there are other, unmeasured, factors at play [17,24].

### 4.1 Study limitations

The biggest limitation of this study is the large amount of missing data on the primary outcome. Due to unforeseen circumstances in the BMT, planned physical fitness measurements which were supposed to be conducted as part of the regular (i.e. non-study) procedures were cancelled by military staff in both the PCP-group as well as in the CON-group. We considered data-imputation techniques and the use of a pattern mixture model since possibly non-responders had lower performance on the run for time than responders. However, since there was no differential dropout, this would not have altered the conclusion. Also, we refrained from any form of data imputation since imputation models would be highly unreliable with this ratio of observed and missing data. Second, whilst adopting a complex system approach, essential factors as training staff support, mindset and health accountability were introduced to all training staff. This may have introduced contamination concerning the non-exercise components of the intervention, reducing the intervention contrast for attrition [25]. Thirdly, due to a constant turnover of military instructors, the PCP was carried out by several instructors. Even though new instructors were consistently briefed about the program, this may have affected intervention fidelity. High turnover of staff is a known barrier to implementation of programs in the military [26]. Also, as a result of staff shortage and difficulties in the housing of recruits, the daily schedule besides sports sessions was spent in various ways, depending on the priorities of the concerned instructors (i.e. foot drill, inspections, weapons training), which may have introduced a certain degree of heterogeneity. Fourthly, lost training days were extracted from the electronic patient records if a military physician prescribed a specific activity restriction, such as five days off from loaded marches. Although guidelines for registering consultation reports [27] and instructive posters which were issued and put on display to remind the physicians of the ongoing study, date of diagnosis and number of restricted training days were not issued completely in all journals. Lost training days were only abstracted from the electronic patient records, in case a military physician explicitly linked a period of absenteeism to an MSI consultation. Therefore, we discarded ‘several days of rest’ in the journals and by that way may have underestimated the number of lost training days due to MSIs. However, this potential information bias will have affected CON and PCP equally, and therefore does not introduce bias in the between group comparison. Lastly, executing a randomised controlled study in the real-life context of BMT posed several challenges, since military training is time and syllabus bound.

### 4.2 Conclusion

In conclusion, our study suggests that improving physical performance to again meet pre-enlistment fitness criteria in deteriorated recruits is feasible, but may be insufficient to significantly reduce the risk of attrition from BMT, perhaps due to common other causes for dropout. It is still not clear how social and mental factors—alongside physical factors—can explicitly be addressed and measured to optimize the effects of integrated injury prevention strategies. Therefore, future research should focus on identifying these factors and their interactions in prospective studies with heterogeneous samples, to help unravel the mechanisms of injury prevention and reducing the risk of dropout from training in recruits.

## Disclaimer

The opinions or assertions contained herein are the private views of the authors and are not to be conceived as official or reflecting the views of the Department of Defence or Dutch government.

## Funding

The views expressed in the submitted article are our own and not an official position of an institution or funder.

This research did not receive any specific grant from funding agencies in the public, commercial, or not-for-profit sectors.

## Financial interests

None of the authors declare competing financial interests.
